# Design of a Curved Shape Photonic Crystal Taper for Highly Efficient Mode Coupling

**DOI:** 10.3390/s21020585

**Published:** 2021-01-15

**Authors:** Reyhaneh Jannesari, Thomas Grille, Cristina Consani, Gerald Stocker, Andreas Tortschanoff, Bernhard Jakoby

**Affiliations:** 1 Institute for Microelectronics and Microsensors, Johannes Kepler University, 4040 Linz, Austria; bernhard.jakoby@jku.at; 2 Infineon Technologies Austria AG, 9500 Villach, Austria; thomas.grille@infineon.com (T.G.); gerald.stocker@infineon.com (G.S.); 3 Silicon Austria Labs GmbH, Europastraße 12, 9524 Villach, Austria; cristina.consani@silicon-austria.com (C.C.); andreas.tortschanoff@silicon-austria.com (A.T.)

**Keywords:** integrated optic, photonic crystal waveguide, taper, mid infrared, non-linear taper, curve shape taper

## Abstract

The design and modeling of a curved shape photonic crystal taper consisting of Si rods integrated with a photonic crystal waveguide are presented. The waveguide is composed of a hexagonal lattice of Si rods and optimized for CO_2_ sensing based on absorption spectroscopy. We investigated two different approaches to design a taper for a photonic crystal waveguide in a hexagonal lattice of silicon rods. For the first approach (type 1), the taper consists of a square lattice taper followed by a lattice composed of a smooth transition from a square to a hexagonal lattice. In the second approach (type 2), the taper consists of a distorted hexagonal lattice. Different shapes, such as convex, concave, and linear, for the curvature of the taper were considered and investigated. The structure of the taper was improved to enhance the coupling efficiency up to 96% at a short taper length of 25 lattice periods. The finite-difference time-domain (FDTD) technique was used to study the transmission spectrum and the group index. The study proves the improvement of coupling using a curved shape taper. Controlling the group index along the taper could be further improved to enhance the coupling efficiency in a wider spectral range.

## 1. Introduction

Photonic crystals (PhCs) are among the most important candidates to confine and guide light through narrowly channeled waveguides and around very tight bends with sizes in the order of optical wavelengths [1,2]. Photonic crystal waveguides (PCWGs) are one of the accepted structures of photonic crystals that are used for realizing integrated optics. Another feature of a PCWG is the low group velocity of the PCWG mode mostly close to the band edge of the dispersion diagram. The group velocity is defined as the derivative of the wavenumber to the frequency. The group velocity for propagating waves crucially affects the efficiency of the light-matter interaction: the lower the group velocity, the higher the intensity of photon-matter interaction [3]. For sensing applications, especially when the concentration of the analyte and its variation is low, efficient interaction between the analyte and light plays an important role; since PCWGs not only improve the sensitivity of the sensor but also reduce the dimension of the device [4]. However, the efficient coupling of an external light source to a narrow PCWG is a challenging problem. In direct butt coupling from a light source to a narrow PCWG, the difference in the modal cross-sectional area causes an abrupt mode transition, which generates a high coupling loss [3]. Also, reflection and scattering from a photonic crystal end cause more loss in direct butt coupling [5]. One of the main reasons for high coupling losses between a dielectric waveguide and a PCWG lies in their intrinsic differences in guiding mechanism and field profile. A waveguide mode in a dielectric waveguide represents a forward-propagation mode, while a mode in a PCWG consists of a superposition of both forward and backward propagation modes. Moreover, in dielectric waveguides, the mode essentially propagates in the region featuring a higher refractive index, whereas for a pillar PCWG, the mode features significant field energy also in the lower refractive index region, which makes it particularly attractive for gas sensing [6,7,8]. On the other hand, pillar-PCWG can exhibit high transmission in wide spectral bands [9]. Therefore, establishing a method for efficient optical coupling to pillar PhC waveguides is essential. A taper can be used to reform a light source to a PCWG mode and thus provides efficient modal coupling between the light source and the narrow PCWG. Often, without a tapering structure, sufficient power that is needed to be used for applications in the integrated optical devices cannot be transmitted to the PCWG.

A photonic crystal taper is a guiding structure for the transmission of electromagnetic waves featuring a funnel shape. It provides smooth mode profile conversion and in addition, it changes the modal properties yielding a higher coupling efficiency. Furthermore, the PhC tapering of a PCWG mode results in a reduction of the group velocity [10,11]. Slow light enhances the light-matter interaction. Most losses of PhC tapers are due to back-reflection, intermodal coupling loss, radiation, and scattering loss. By optimizing the shape and the length of the taper, the back-reflection loss can be minimized [5]. Theoretically and experimentally, the coupling between pillar PhC and wire waveguides is already investigated [9,12,13]. Most reliable structures based on adiabatic transition consist of a long coupler and sharp tips [14]. However, demands for miniaturization in integrated optics and the limited critical dimensions of fabrication processes make them undesired.

A photonic crystal taper can be obtained by progressive variation of the crystal geometry. Hence, light traveling through the taper will experience adiabatic mode transformation. An adiabatic transmission can occur if the operating mode is propagating (nonevanescent) and guided at every point in the taper [15]. The chirping of the PhC geometry can be done by two approaches, lattice distortion of a properly structured crystal arrangement or gradual change in the size of PhC elements [6,11]. The size changing needs highly accurate lithography to control the element size accurately. Furthermore, it suffers from high radiation losses and back reflection at the uneven sidewall of the taper [5]. In this work, the first approach (lattice distortion) is chosen to design a low loss coupling device. In this approach, avoiding sharp tips or small elements together with a short length is considered. Such a system is fully compatible with advanced Si-based fabrication technologies such as Complementary metal-oxide-semiconductor (CMOS) and micro-electro-mechanical systems (MEMS), so the fabrication process is manufactural.

In this paper, we present a design of a curve shape PhC taper to improve the mode coupling efficiency from an optical fiber to a pillar PCWG with a hexagonal lattice at a short taper length. Moreover, two complementary curve shapes, with hexagonal and square lattices, are investigated, and their reflection losses are compared. The slow light with a high group index in PCWGs limits the bandwidth of slow light hence disturbs some practical applications. We discuss and compare the transmission spectrum and the group index of designed tapers.

## 2. Photonic Crystal and Taper Structure

**Figure 1:**
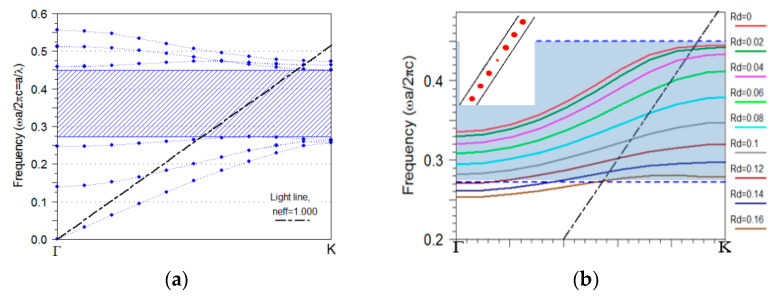
(**a**) TE dispersion band diagrams of a PhC of silicon rods in a hexagonal lattice with r=0.2a. The shaded area presents the photonic bandgap and the dash-dotted black line shows the light line. Modes below the light line are vertically confined into the PhC, (**b**) dispersion diagram of the PCWG modes with different radii inside the bandgap, in which the periodic supercell for simulation is shown in the inset.

As a basis, a two dimensional (2D) hexagonal PhC of silicon rods is considered for the design of the PCWG and the taper. The PhC lattice consists of high index rods (n=3.4247, e.g., poly-silicon) in air (n=1). The radius of the rods is taken as r=0.2a, where r is the radius of the rod and a is the lattice constant. This ratio is used to obtain a large TE (dominant electric field component parallel to the rods) bandgap for the hexagonal lattice of silicon rods. The resulting photonic bandgap turns out to be in the range of 0.272–0.451 in unit of the normalized frequency (a/λ). Figure 1a presents the band structure of the PhC. The shaded area indicates the photonic bandgap. The plane wave expansion (PWE) method was used for this calculation [16]. The resolution used is 32 grid points per basis vector of unit cell. The parameters of the PhC are selected in such a way that provides a bandgap around 4.26 µm. We analyze the detection of CO_2_ based on its mid-IR absorption peak at 4.26 µm as a case study. The mid-infrared (MIR) spectral window contains characteristic absorption lines of many gases such as CO_2_, CH_4_, CO, etc., which is why this region is referred to as the “figureprint” region [17]. Among different sensing methods, infrared absorption spectroscopy is often considered preferable. The infrared absorption spectroscopy has high sensitivity and selectivity associated with the characteristic spectral absorption pattern of an analyte [18]. The PCWG of Si rods used in this work can enhance the light–matter interaction, which leads to improved sensitivity, which is particularly important when sensing dilute gases [19].

**Figure 2:**
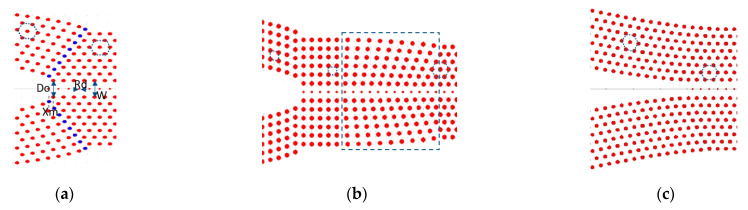
(**a**) Schematic of a PhC convex taper with a hexagonal lattice, blue dots indicate an extra column of silicon rods which added to the lattice of the hexagonal taper; (**b**) Schematic of a PhC convex taper with square lattice followed by a smooth transition from square to the hexagonal lattice, indicated with the dashed square; (**c**) Schematic of a PhC concave taper with hexagonal lattice.

The radius of rods in one row of photonic crystal ($R_{d}$) was altered to create a photonic crystal waveguide (PCWG). The dispersion diagram of the PCWGs with different radii $R_{d}$ is displayed in Figure 1b. The supercell for PCWG mode calculation has a lateral length of one row of Si rods in the x-direction and seven rows of Si rods in the y-direction (inset Figure 1b), which is large enough to avoid the coupling between the adjacent parallel waveguides. Changing the radii of pillars in PCWG from zero to higher values shifts the waveguide mode to lower energy. Therefore, this parameter is used to tune the frequency of PCWG mode to the desired frequency range. The $R_{d}=0.06a$ is chosen for this study because the PCWG is designed for CO_2_ sensing [19,20] Figure 2a,b presents a schematic view of the PCWG.

The photonic crystal taper operates within a photonic bandgap to diminish the loss due to the coupling to the bulk of the photonic crystal. A mode with a frequency inside the bandgap cannot propagate inside the PhC structure. Therefore, the PhC functions as a mirror and reduces the lateral leakage loss in the PhC taper. Moreover, as the width of the photonic crystal taper narrows towards one end along its axis, the number of modes also decreases, which leads to a smooth mode profile conversion, yielding a higher coupling efficiency to a single-mode PCWG.

**Figure 3:**
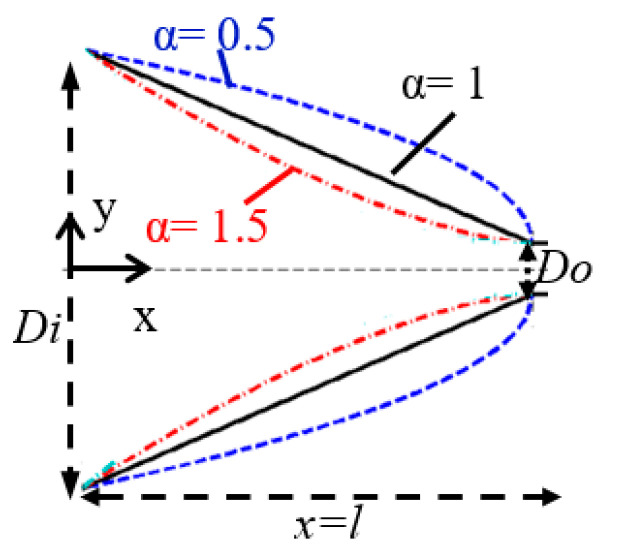
Various taper shapes for different values of α. The corresponding shape of taper for α=0.5, 1 and 1.5 is convex, linear and concave, respectively.

In this work, the photonic crystal taper is depicted by deforming and cleaving the photonic crystal lattice according to the following equation [4]

(1) $$y=D_{i}+(D_{i}-D_{o})\left[{\left(1-\frac{x}{l}\right)}^{\alpha }-1\right]\;$$

where $D_{i}$ and $D_{o}$ are the input and output width of the taper, respectively (Figure 3). Variables x and y define the horizontal and lateral position of the center of each rod with respect to the input of the taper, respectively. The parameter l is the total length of the taper and α defines the shape of the taper as follows: α<1 convex taper, α=1 linear taper, and α>1 concave taper. The innermost row of the rods is spotting according to Equation (1) to shape the taper.

The concept of taper with a square lattice to control the coupling of light has been already studied by Khoo et.al. [4]. However, the concept of a curved shape taper with a hexagonal lattice has not been studied so far to the best of our knowledge.

**Figure 4:**
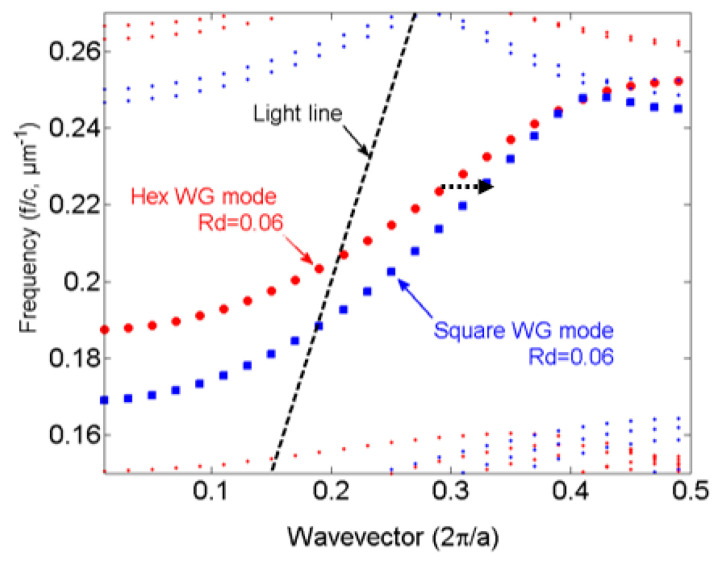
Dispersion diagram of PCWGs with hexagonal lattice (red dot) and square lattice (blue dots), respectively.

For our application, a PCWG with a hexagonal lattice is needed [21]. Therefore, we have two different approaches to design a taper for a PCWG in a hexagonal lattice of silicon rods. For the first approach (type 1), the taper consists of a square lattice of silicon rods which followed by a lattice composed of a smooth transition from a square symmetry to a hexagonal symmetry. In the second approach (type 2), the taper consists of a distorted hexagonal lattice, this taper does not need the transition lattice. Schematic views of the two taper types are presented in Figure 2a,b. The total length of the taper type 1 is longer than that of type 2. The red (blue) dots in Figure 4 show the dispersion band diagram of the PCWG with $R_{d}=0.06a$ in a PhC with hexagonal (square) lattice symmetry. As can be seen, by the black dotted arrow, taper type 1 suffers from an additional loss because of the mode mismatching between the hexagonal PCWG and the square PCWG. Therefore, the type 2 taper was considered for the optimization of its geometrical parameters for the enhancement of the taper transmission at *λ* = 4.26 µm.

## 3. Optimization of Geometrical Parameters

To simulate the transmission, incident light is launched into the PhC taper and its response at the output of the taper is observed. For this calculation, the software package RSoft based on the finite-difference-time-domain (FDTD) method is used [16]. The incident light source, which is a single-mode fiber, is assumed to provide a Gaussian beam mode distribution. The waveguide consists of a row of Si rods with $R_{d}=0.06a$ in a hexagonal lattice, which is optimized for the CO_2_ sensing based on absorption spectroscopy (*λ* = 4.26 µm). The reflected and transmitted fields are calculated behind the source and at the output of the PCWG, respectively. Poynting vector is used to convert the collected field to power and normalized with the incident power to obtain the normalized transmission for different tapers.

**Figure 5:**
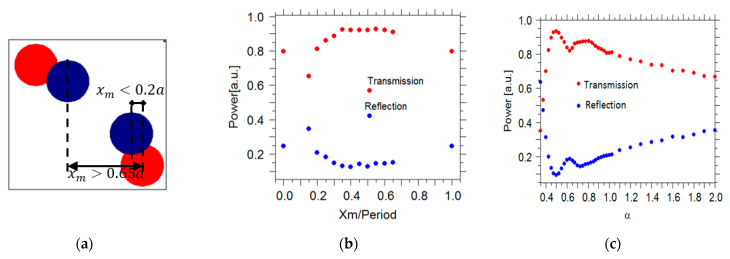
(**a**) A Schematic illustration to show how Si rods in the extra column (dark blue circle) overlap with Si rods (red circle) of the lattice. Transmission (red dots) and reflection (blue dots) (**b**): as function of $x_{m}$ for α=0.5 (**c**) as function of α, for PCWG with $R_{d}=0.06a$ respectively.

According to Equation (1), the shape of the taper is changed and the power transmitted to the PCWG for different values of α is calculated. However, the width of the convex taper (α < 1) features a rapid change when approaching the end of the taper. Therefore, close to the taper-end, the vertical distance between rods increases, which causes an in-plane radiation loss (leakage). Hence, light traveling through the taper will experience a non-adiabatic mode transformation. To address this issue an extra column of Silicon rods was added to the lattice to reduce lateral radiation loss. The extra column of silicon rods is positioned within a lattice period *a*, which is presented with blue dots in Figure 2a. The parameter $x_{m}$ defines the position of the most inner rod of the extra column along the x-axis. Figure 5b shows the transmission efficiency of the convex taper with α=0.5 versus$\;x_{m}$. Without the extra column ($x_{m}=0,1$) the maximum transmission is around 65%. However, by adding the extra column, the transmission efficiency improves to more than 95%, which verify reduced radiation loss. Each Si rod has a radius of R = 0.2*a*. For $x_{m}<0.15a$ and $x_{m}>0.65a$ the silicon rods in the extra column overlap with the silicon rods of the lattice as shown in Figure 5a. Consequently, some defect centers with different shapes are introduced into the lattice, which causes some anomaly radiation or reflections. Hence, the acceptable range for parameter $x_{m}$ is $0.15a<x_{m}<0.65a$.

**Figure 6:**
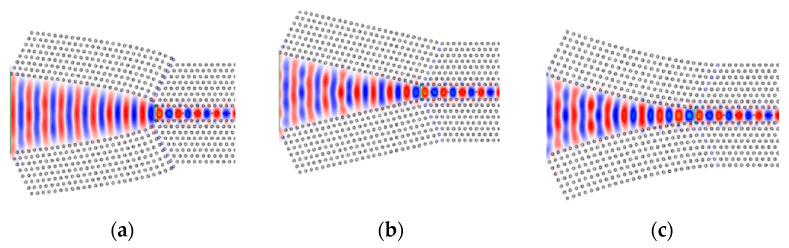
Calculated electric field distribution for a: (**a**) convex taper; (**b**) linear taper; (**c**) concave taper.

In Figure 5c the transmission efficiency for different taper shapes, represented by α, is presented. Owing to the indentation shape of the concave taper, at the beginning of the taper the width of the concave taper is decreasing at a faster rate compared to the linear and convex tapers, which caused some reflection loss. Hence, the transmission efficiency for α>1 is less than that for the linear taper (α=1). The characteristics for the convex taper (α<1) show a smaller slope in the beginning, which approaches the adiabatic conversion of the mode. This results in higher transmission than in the case of a linear taper. The taper with α=0.5 yields the maximum transmittance efficiency of 0.96%. This observation shows that because of the diminished losses, the coupling is more effective at the convex taper. The convex taper with α=0.5 and the PCWG with $R_{d}=0.06$ are chosen for the further optimization of the taper. Figure 6 shows the normalized electric field distribution of the incident light through the different taper designs.

**Figure 7:**
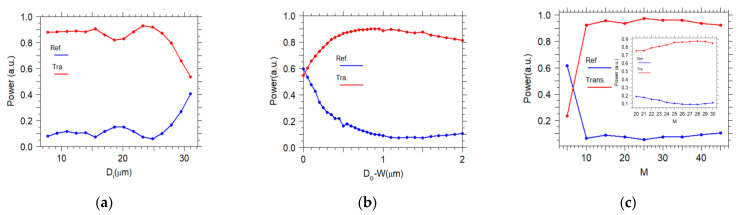
Transmission and reflection change as a function of (**a**) input width, (**b**) output width, (**c**) taper length, for a convex taper at λ=4.26 µm, respectively.

In the following, the effect of the input width, the output width, and the length of the taper on the transmission is investigated. The main function of a photonic crystal taper is the transformation from a large cross-section fiber to a small cross section PCWG [11]. Figure 7a presents the transmission power versus the input width at a fixed length and the output width. For input widths between 7 and 15 µm, the transmitted power is almost constant. However, for larger the input width, the transmitted power has some fluctuations because, at a given length and output width, there is a fast change in the width of the convex taper. Therefore, more back-reflection loss occurs. Considering conventional fibers, an input width of 15 µm is a feasible choice for this work.

In Figure 7b, the transmission power versus $D_{o}-W$ is displayed, where $D_{o}$ is the output width of the taper and $W=\sqrt{3}a$ is the width of the PCWG. It can be seen from Figure 7b that expanding the output width of the taper to values larger than the input width of the PCWG $\left(D_{o}>W\right)$ enhances the transmission power. It is important to note that the FDTD calculation confirms that by expanding the output width of the taper, no frequency shift occurs to the PCWG mode. As evident from Figure 7c, varying the length of the taper improves the mode-conversion efficiency to increase the coupling efficiency. Here, the factor M is the number of unit-cells of the PhC along the taper, which means taper length is l=Ma. According to this diagram, for the taper with a longer length, the slope of the taper is more moderate. Thus the adiabatic regime is approached, resulting in more effective modal coupling, at a fixed input and output width of the taper. For the length larger than 25a(l>25a) the transmission is almost constant (inset Figure 7c).

The same optimization study has been done for the taper with square lattice (type 1). However, the result shows always better transmission efficiency for the hexagonal taper (type 2).

Future work will address the fabrication of the device. Nevertheless, we describe here the potential fabrication process that could be considered. The first step in the fabrication process is the mask processing using the deep ultraviolet (DUV) lithography method to pattern the photonic crystal cavity mask on the resist. After that, a thin layer of a metal, such as gold or chromium, is deposited on the sample. By performing a lift-off process, the PhC pattern is created on the metal as a hard mask. The top Si layer is vertically etched by the deep reactive ion etching process (DRIE, “Bosch process”). Finally, the metal layer is removed in a wet etch process.

## 4. Investigation of Transmission Spectrum

Let us finally present the effect of the taper shape on the spectrum of the transmitted power. FDTD method is used to calculate the transmittance spectrum. A pulse excitation is used to measure the frequency response of each structure. The pulse consists of a Gaussian envelope function multiplying a sinusoidal carrier. The monitor’s locations are taken behind the input signal to calculate the reflection spectrum from the taper and at the output of the taper to calculate the output field passing through the taper. For all calculations the resolution is set to a rectangular grid of a/32, where a represents the lattice constant. The boundaries of the simulation area are surrounded by perfectly matched layers (PML).

**Figure 8:**
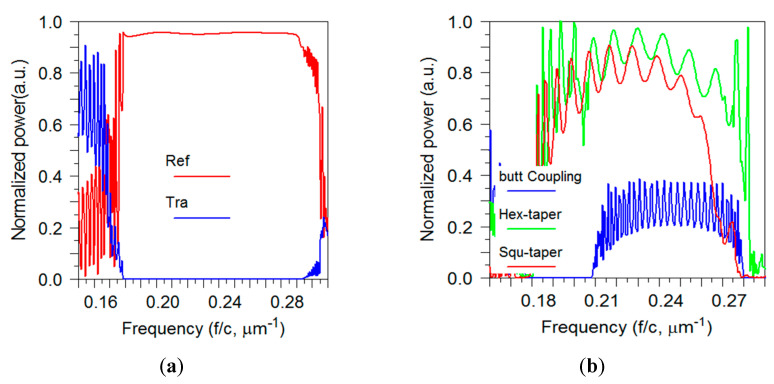
(**a**) Transmittance and reflectance spectrum of the PhC with hexagonal lattice calculated with FDTD method. The bandgap has a good agreement with the band structure calculated with PWE method (Figure 1a). (**b**) Transmission spectrum for the butt coupling into the PCWG (blue), the taper coupling with square lattice (red) and the taper coupling with hexagonal lattice (green).

In the first place, a pulse is launched into the bulk PhC. Figure 8a presents the transmittance and reflectance of the bulk PhC. In the frequency range of 0.178–0.294 (1/λ) µm^−1^ the transmission is highly diminished, which is expected since the pulse is placed in the bandgap. The normalized frequency is in the range of 0.276–0.455(a/λ) which has a good agreement with the dispersion diagram presented in Figure 1a. This bandgap is our region of interest because working in this region reduces the coupling loss due to the bulk of photonic crystals [11]. The butt coupling together with the transmission spectrum of two types of tapers introduced in Section 2 with α=0.5 is shown in Figure 8b. The two tapers have hexagonal and square lattice symmetry, respectively. For a correct comparison, all three structures have a compatible length. The butt coupling has an average transmission efficiency of 25%, whereas the average transmission efficiency for the taper with square lattice is changing between 60% and 80% over the frequency range of 0.18–0.26 (1/λ) µm^−1^. However, the transmission efficiency for the taper with hexagonal lattice is changing between 60–90% in the frequency range of 0.18–0.28 (1/λ) µm^−1^ which is a significant improvement. As can be seen, the hexagonal taper has a wider transmission frequency range than others, besides the transmission is always higher for the hexagonal taper in all frequency range. This strongly proves the advantage of using the hexagonal array taper with respect to the square array taper.

**Figure 9:**
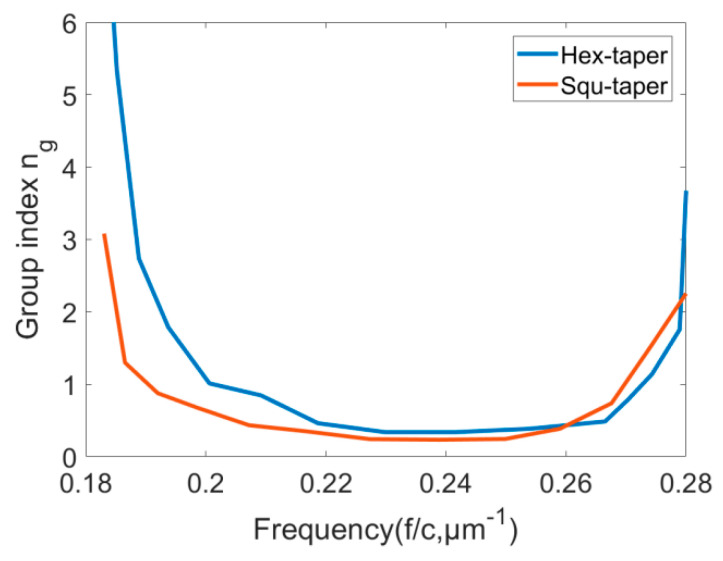
Deduced group index curves ($n_{g}$) vs. wavelength for different tapers.

Fluctuations in the spectrums indicate some Fabry–Perot oscillations, which are due to the multiple reflections from the mirror cavity formed by the sides of the taper. Furthermore, the fluctuations are observed as a consequence of the “multipath” interferences caused by coupling back and forth between the multiple modes [5]. Figure 8b shows that the oscillation period is not constant which suggesting a strongly dispersive character. To figure out the applicability of tapers, as coupling devices, the dispersion characteristics of the tapers also are investigated [12]. The tapering changes the group velocity of a mode. The group velocity of a PhC mode at a particular wavelength can be determined from the slope of the dispersion diagram. The group velocity is expressed as $v_{g}=c/n_{g}=\partial \omega /\partial k$ [13]. As a result of tapering, the group velocity of the guided mode coupled from the fiber could slow down gradually over the length of the taper until it enters the slow light waveguide. Due to a smaller difference in group velocity between modes in a taper coupling loss is reduced. The curved shape photonic crystal tapers control the dispersion to achieve optimized coupling. The following relation is used to calculate the group index, $n_{g}$, [22]:

(2) $$n_{g}=\frac{\lambda^{2}}{2l\Delta \lambda }$$

where l represents the coupling length. The transmission spectrum versus the wavelength regime is used and a Lorentzian peak is fitted to each Fabry-Perot peak to deduce the parameters λ and Δλ. Figure 9 summarizes the deduced $n_{g}$ for two types of tapers as a function of normalized frequency (1/λ). The slope of the group index curve shows the group velocity dispersion (GVD). A mode with low group velocity (namely, high group index) is usually accompanied by large GVD, which would severely limit the bandwidth of slow light, deforming optical pulses [23]. Generally, the wavelength range over which the group index remains constant is considered as the useful bandwidth of the device, and in many papers, the group index is considered as constant when the fluctuation of it is within a ±10% range [24,25]. In the lower frequency range, the slope of $n_{g}$ for the square taper is smaller as compared to the hexagonal taper resulting in better transmission. However, the useful bandwidth range of the square taper is 0.207–0.259(1/λ) µm^−1^, and the hexagonal taper is 0.2187–0.2665(1/λ) µm^−1^, which shows that the square taper has a wider useful bandwidth than the hexagonal taper. In the case of slow light, the confinement of light is increased, which leads to high photon density, in other words, effective light-matter interaction is enhanced as $n_{g}$ becomes large [22]. In the marginal region of the spectrum, the group index has higher variations for the hexagonal taper, which make it particularly suitable for sensing applications utilizing low group velocity [20,21].

## 5. Conclusions

In this work, a novel taper structure on a two-dimensional PhC consisting of a hexagonal lattice of silicon rods is designed and validated through computational simulation for the coupling of a fiber mode to the PCWG. The dispersion characteristics of the tapers with hexagonal and square lattice are investigated to find out their suitability as coupling devices. The adiabatic mode conversion in the hexagonal PhC taper provides a more efficient coupling by smoothly shifting the dispersion characteristics. The proposed PhC taper combines two features. It can not only reduce the mode field mismatch but also transform between the forward propagating mode and the bi-directional propagating mode. The FDTD simulation results prove that the hexagonal PhC taper has high transmission efficiencies of up to 90% in a wide frequency range and can convert the mode profile smoothly. Studying the GVD and the spectrum of a given taper shows that the transmission features are wavelength dependent. This taper also can be utilized in the slow light region, which has applications in devices based on the interaction between light and matter. The hexagonal lattice taper is optimized for *λ* = 4.26 µm to achieve transmission efficiency, which is more than 96%. The taper has a length of 25a for direct coupling between an optical fiber and the PCWG.

## Data Availability

Not applicable.

## References

[1] Dutta H.S., Goyal A.K., Srivastava V., Pal S. (2016). Coupling light in photonic crystal waveguides: A review. Photonics Nanostruct. Fundam. Appl..

[2] Chutinan A., Noda S. (2000). Waveguides and waveguide bends in two-dimensional photonic crystal slabs. Phys. Rev. B.

[3] Pergande D., von Rhein A., Geppert T.M., Wehrspohn R.B. (2009). Coupling schemes for low-group velocity photonic crystal devices. J. Comput. Theor. Nanosci..

[4] Mekis A., Joannopoulos J.D. (2001). Tapered couplers for efficient interfacing between dielectric and photonic crystal waveguides. J. Lightwave Technol..

[5] Khoo E., Liu A., Wu J. (2005). Nonuniform photonic crystal taper for high-efficiency mode coupling. Opt. Express.

[6] Liua J., Gaoa D., Zhou Z. (2006). Photonic Crystal Taper for Efficient Coupling and Smooth Mode Profile Conversion. Optics Valley of China International Symposium on Optoelectronics.

[7] Jannesari R., Ranacher C., Consani C., Lavchiev V., Grille T., Jakoby B. (2016). High-Quality-Factor Photonic Crystal Ring Resonator with Applications for Gas Sensing. Procedia Eng..

[8] Ao X., Liu L., Wosinski L., He S. (2006). Polarization beam splitter based on a two-dimensional photonic crystal of pillar type. Appl. Phys. Lett..

[9] Tokushima M., Arakawa Y. (2017). Double-stage guided-mode converter for pure TM-mode guiding in pillar photonic-crystal waveguide devices. Opt. Express.

[10] Vlasov Y.A., McNab S.J. (2006). Coupling into the slow light mode in slab-type photonic crystal waveguides. Opt. Lett..

[11] Joshi N., Janyani V. (2009). Efficient coupling of light into photonic crystal waveguides using chirped photonic crystal tapers. J. Opt..

[12] Vercruysse D., Sapra N.V., Su L., Vuckovic J. (2020). Dispersion Engineering With Photonic Inverse Design. IEEE J. Sel. Top. Quantum Electron..

[13] Assefa S., Bienstman P., Rakich P., Johnson S.J., Joannopoulos J.D., Petrich G.S., Kolodziejski L.A. (2003). Taper structures for coupling into photonic crystal slab waveguides. J. Opt. Soc. Am. B.

[14] Xu Y., Lee R.K., Yariv A. (2000). Adiabatic coupling between conventional dielectric waveguides and waveguides with discrete translational symmetry. Opt. Lett..

[15] Johnson S.G., Bienstman P., Skorobogatiy M.A., Ibanescu M., lidorikis E., Joannopoulos J.D. (2002). Adiabatic theorem and continuous coupled-mode theory for efficient taper transitions in photonic crystals. Phys. Rev. E Stat. Phys. Plasmas Fluids Relat. Interdiscip. Top..

[16] RSoft’s Photonic Design Suite. Version Synopsys RSoft 2019.09. RSoft’s Photonic Design Suite. https://www.synopsys.com/photonic-solutions.html.

[17] Zhao Y., Zhang Y., Wang Q. (2011). Research advances of photonic crystal gas and liquid sensors. Sens. Actuators B Chem..

[18] Pergande D., Geppert T.M., Rhein A., Schweizer S.L., Wehrspohn R.B., Moretton S., Lambrecht A. (2011). Miniature infrared gas sensors using photonic crystals. J. Appl. Phys..

[19] Jannesari R., Abasahl B., Grille T., Jakoby B. (2018). Hybrid Photonic Crystal-Surface Plasmon Polariton Waveguiding System for On-Chip Sensing Applications. Proceedings.

[20] Jannesari R., Grille T., Jakoby B. Gas sensing with a high-quality-factor photonic crystal ring resonator. Proceedings of the SPIE Optics + Optoelectronics.

[21] Jannesari R., Ranacher C., Consani C., Grille T., Jakoby B. (2017). Sensitivity optimization of a photonic crystal ring resonator for gas sensing applications. Sens. Actuators A Phys..

[22] Notomi M., Yamada K., Shinya A., Takahashi J., Takahashi C., Yokohama I. (2001). Extremely Large Group-Velocity Dispersion of Line-Defect Waveguides in Photonic Crystal Slabs. Phys. Rev. Lett..

[23] Lau W.T., Fan S. (2002). Creating large bandwidth line defects by embedding dielectric waveguides into photonic crystal slabs. Appl. Phys. Lett..

[24] Zhao Y., Zhang Y.N., Wang Q., Hu H. (2015). Review on the Optimization Methods of Slow Light in Photonic Crystal Waveguide. IEEE Trans. Nanotechnol..

[25] Wan Y., Fu K., Li C.H., Yun M.J. (2013). Improving slow light effect in photonic crystal line defect waveguide by using eye-shaped scatterers. Opt. Commun..

